# The CD39/CD73/Adenosine and NAD/CD38/CD203a/CD73 Axis in Cutaneous T-Cell Lymphomas

**DOI:** 10.3390/cells14040309

**Published:** 2025-02-19

**Authors:** Liyun Lin, Gabriele Roccuzzo, Yuliya Yakymiv, Sara Marchisio, Erika Ortolan, Ada Funaro, Rebecca Senetta, Valentina Pala, Martine Bagot, Adèle de Masson, Maxime Battistella, Emmanuella Guenova, Simone Ribero, Pietro Quaglino

**Affiliations:** 1 Laboratory of Immunogenetics, Department of Medical Sciences, University of Turin, 10126 Turin, Italy; liyun.lin@unito.it (L.L.); yuliya.yakymiv@unito.it (Y.Y.); sara.marchisio@unito.it (S.M.); erika.ortolan@unito.it (E.O.); ada.funaro@unito.it (A.F.); 2 Section of Dermatology, Department of Medical Sciences, University of Turin, 10126 Turin, Italy; gabriele.roccuzzo@unito.it (G.R.); v.pala@unito.it (V.P.); pietro.quaglino@unito.it (P.Q.); 3 Pathology Unit, Department of Oncology, University of Turin, 10126 Turin, Italy; rebecca.senetta@unito.it; 4 Dermatology Department, Saint-Louis Hospital, AP-HP, Université Paris Cité, Inserm U976, 75010 Paris, France; martine.bagot@gmail.com (M.B.); adele.demasson@aphp.fr (A.d.M.); 5 Department of Pathology, Hôpital Saint-Louis, AP-HP, Université Paris Cité, Inserm U976, 75010 Paris, France; maxime.battistella@aphp.fr; 6 Department of Dermatology, Lausanne University Hospital (CHUV) and Faculty of Biology and Medicine, University of Lausanne, 1007 Lausanne, Switzerland; emmanuella.guenova@unil.ch; 7 University Institute and Clinic for Immunodermatology, Medical Faculty, Johannes Kepler University, 4020 Linz, Austria

**Keywords:** cutaneous T-cell lymphoma, Sézary syndrome, adenosine, CD39, CD73, CD38

## Abstract

Cutaneous T-cell lymphoma (CTCL), characterized by malignant T-cell proliferation primarily in the skin, includes subtypes such as mycosis fungoides (MF) and Sézary syndrome (SS). The tumor microenvironment (TME) is central to their pathogenesis, with flow cytometry and histology being the gold standards for detecting malignant T cells within the TME. Alongside emerging molecular markers, particularly clonality analysis, these tools are indispensable for accurate diagnosis and treatment planning. Of note, adenosine signaling within the TME has been shown to suppress immune responses, affecting various cell types. The expression of CD39, CD73, and CD38, enzymes involved in adenosine production, can be elevated in MF and SS, contributing to immune suppression. Conversely, the expression of CD26, part of the adenosine deaminase/CD26 complex, that degrades adenosine, is often lost by circulating tumoral cells. Flow cytometry has demonstrated increased levels of CD39 and CD73 on Sézary cells, correlating with disease progression and prognosis, while CD38 shows a variable expression, with its prognostic significance remaining under investigation. Understanding these markers’ roles in the complexity of TME-mediated immune evasion mechanisms might enhance diagnostic precision and offer new therapeutic targets in CTCL.

## 1. Introduction

**Figure 1:**
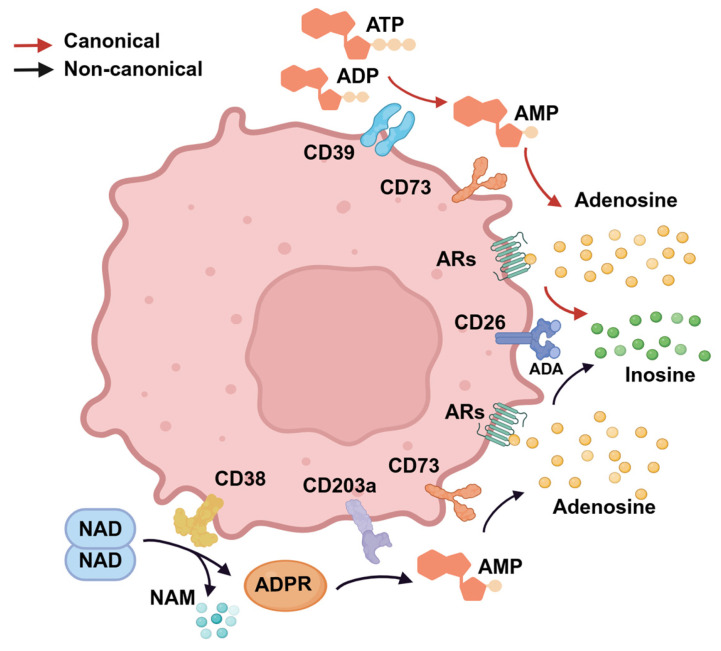
Adenosine axes. In the canonical pathway (red arrows), the extracellular ATP is converted into adenosine by continuous hydrolysis of CD39 and CD73. In the non-canonical pathway (black arrows), NAD is initially metabolized by CD38 into NAM and ADPR. ADPR can be hydrolyzed into AMP under the action of CD203a. Then, CD73 catalyzed AMP to generate adenosine. The adenosine generated by these two pathways mediates its immunoregulatory functions by binding to one of ARs. The ADA/CD26 complex catalyzes the deamination of adenosine to inosine, thus reducing the interstitial adenosine levels. NAD, nicotinamide adenine dinucleotide; NAM, nicotinamide; ADPR, ADP-ribose; ARs, adenosine receptors; ADA, adenosine deaminase.

Cutaneous T-cell lymphomas (CTCL) represent a heterogeneous group of non-Hodgkin lymphomas characterized by the proliferation of malignant T cells primarily in the skin. According to the WHO-EORTC update, the proportion of CTCL among primary cutaneous lymphomas is up to 75–80% in the Western world [1]. Mycosis fungoides (MF) represents the most common form of CTCL, whilst Sézary syndrome (SS), currently classified in the class/family of mature T-cell leukemias, is distinguished by the presence of erythroderma, lymphadenopathy, and the dissemination of malignant T cells (Sézary cells) in the peripheral blood [2]. Recently, new entities have been identified, and the international classification has undergone significant updates [1,2,3]. While the exact pathogenesis of CTCL and SS remains incompletely elucidated, several reports indicate that the tumor microenvironment (TME) is involved in their development and progression [4,5]. The TME can help tumor cells evade immune surveillance by enhancing the immunosuppressive state through a variety of mechanisms [6]. For example, malignant T cells often express high levels of immune checkpoint molecules such as PD-1 and CTLA-4, which inhibit the activity of effector T cells, thus limiting anti-tumor immune responses [7,8]. Tumor cells also secrete immune-suppressive cytokines, such as IL-10 [9,10,11], and can activate TGF-β [12], forming a network of immune-suppressive factors, further creating a conducive environment for tumor growth. In addition to the mentioned mechanisms, the TME can be modulated by the adenosine-signaling pathway. Tumor cells enhance adenosine production by upregulating key enzymes such as CD39 and CD73. Adenosine binds to adenosine receptors (ARs) on the surface of a variety of immune cells and, through the generation of cAMP, can directly regulate tumor proliferation, survival, adhesion, and invasion [13]. In immune cells, adenosine greatly hampers immune effector cell functions and may be involved in mediating T-cell exhaustion (Figure 1) [14].

Adenosine is mainly produced through the CD39/CD73/adenosine canonical pathway. The extracellular nucleotide enzyme CD39, also known as ecto-nucleoside triphosphate diphosphohydrolase 1 (E-NTPDase1), catalyzes the hydrolysis of ATP and ADP to AMP. CD39 is primarily expressed in the spleen, thymus, and lung tissues, predominantly by vascular endothelial cells, regulatory T cells (Tregs), monocytes, and a subset of T-helper 17 cells (Th17) [15,16,17]. It is also highly expressed in several tumors, including ovarian cancer, colorectal cancer, multiple myeloma, and lymphoma [18,19,20,21]. CD39 comprises 510 amino acids, containing 11 cysteine residues and 7 potential N-linked glycosylation sites, along with 2 transmembrane domains. The transmembrane domains feature five highly conserved sequence motifs that mediate the enzyme’s nucleotidase activity [22,23]. CD73, another extracellular nucleotide enzyme also known as ecto-5′-nucleotidase (NT5E), further catalyzes the conversion of AMP to adenosine [24]. CD73 is a glycosylphosphatidylinositol (GPI)-anchored glycoprotein that functions as a dimer on the plasma membrane. It is broadly expressed in various tissues, including colon, brain, kidney, liver, lung, and heart tissues, and is present in both immune and non-immune cells [25]. It has been demonstrated that CD73 is highly expressed in multiple solid tumors, including melanoma, colorectal cancer, bladder cancer, and ovarian cancer, and plays a pivotal role in various physiological and pathological processes [16,26]. In the TME, cellular stress or necrosis leads to the release of ATP, enhancing its concentration in the extracellular space. ATP is hydrolyzed by CD39 to AMP with the transient generation of free ADP. Then, CD73 dephosphorylates AMP to produce adenosine. In addition to the CD39/CD73/adenosine axis, adenosine can also be generated from nicotinamide adenine dinucleotide (NAD) through the non-canonical adenosinergic CD38/CD203a/CD73 pathway [27]. CD38, also referred to as cyclic ADP-ribose hydrolase, is present on the surface of various immune cells, including CD4+ and CD8+ T cells, B cells, and natural killer cells [28]. It is primarily distributed in the bone marrow, lymph nodes, thymus, spleen, and tonsils [29]. CD38 plays a critical role in the pathogenesis of various lymphoid malignancies, such as mantle cell lymphoma, chronic lymphocytic leukemia, and multiple myeloma [30,31,32]. CD38 metabolizes extracellular NAD, producing nicotinamide (NAM) and ADP-ribose (ADPR) that can be hydrolyzed by CD203a (ectonucleotide pyrophosphatase/phosphodiesterase 1, ENPP1) into AMP, which is then converted to adenosine by CD73, which represents the common link between the two adenosinergic pathways [33,34]. The collaborative action of these two pathways synergistically catalyzes the generation of adenosine. In physiological conditions, the extracellular adenosine is irreversibly deaminated into inosine by the adenosine deaminase (ADA)/CD26 complex [35].

Currently, Sézary cells’ detection essentially relies on the loss of expression of the CD7 and/or CD26 antigen rather than on the aberrant expression of specific markers [26,27]. Recent studies highlight the abnormal expression of CD39, CD73, and CD38 in CTCL, although their diagnostic potential needs further exploration. This review aims to assess these markers’ utility in Sézary cells’ identification and the diagnosis of selected subtypes of CTCL, offering new diagnostic tools.

## 2. Literature Search

**Figure 2:**
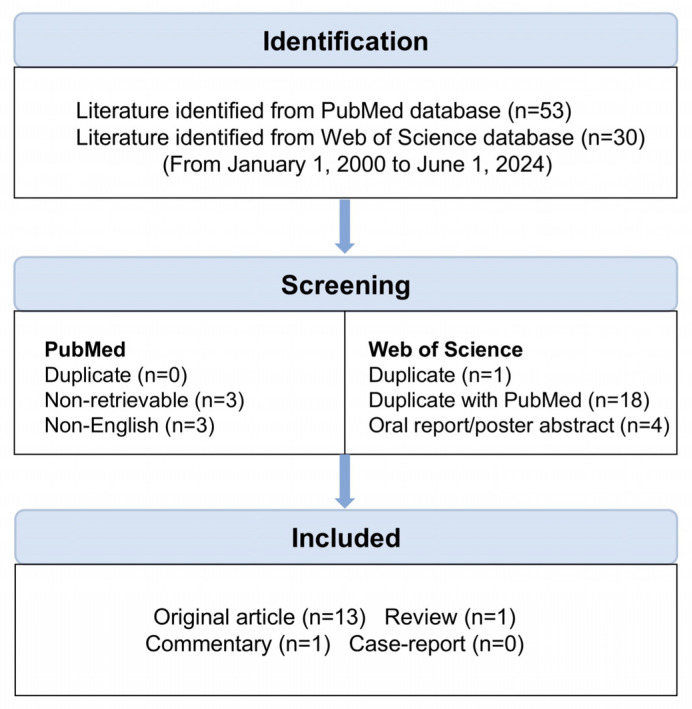
Search process. We performed a literature search using PubMed and Web of Science, covering publications from 1 January 2000 to 1 June 2024. Duplicate entries, non-retrievable ones, and non-English literature were excluded from the search results. The retrieved literature was categorized into four sections: original article, review, commentary, and case report. Each publication was meticulously reviewed to ascertain its relevance to the search terms and irrelevant articles were excluded from the final analysis. Finally, 13 original articles, 1 review, and 1 commentary were included.

The literature search was conducted on 1 June 2024, across two major databases: PubMed and Web of Science. Our search terms comprised [“CD39” OR “CD73” OR “CD38”] AND [“CTCL” OR “Sézary syndrome”]. To ensure comprehensive coverage, we employed synonyms for each search term obtained from the MeSH database. Ultimately, 15 studies were included, mainly original articles, with one review (No. 4) and one commentary (No. 13) (Figure 2, Table 1).

## 3. Discussion

### 3.1. Expression of CD39, CD73, and CD38 in CTCL Blood Samples

The detection of tumor cells in peripheral blood has a key role in the diagnosis and management of SS. Blood staging (B0–B2) is a part of the diagnostic process and relevant for the prognosis in CTCL. Blood disease stages currently as B0 (<5% tumor cells or <250/μL), B1 (5% or <1000/μL), and B2 (≥1000/μL) [51]. The B staging correlates with tumor burden, with higher stages indicating a worse prognosis. The accurate identification and differentiation of Sézary cells from normal T cells is crucial for patient management [52]. In this instance, flow cytometry has replaced manual counting and electron microscopy to enable the immunophenotypic analysis of T-cell subtypes [53]. Flow cytometry allows both qualitative (abnormal T-cell population identification) and quantitative analysis (Sézary cells count) [54]. Clinically, CD4+CD7− and/or CD4+CD26− cells are often equated with Sézary cells, supported by studies assessing T-cell clonality [51]. However, up to 40% of reactive CD4+ T cells can lose CD7, and 30% can lose CD26 [55], whilst around 15% of SS patients may have CD26+ subpopulations [36]. The TCR Vβ flow cytometrical analysis can assess clonality but it is laborious, detects only 70% of cases, and comes with a relatively high price for a conventional cytometry [56,57]. As an alternative, the anti-TRBC1 antibody (JOVI-1 clone) against one of the two mutually exclusive T-cell receptor β-chain constant domains was proposed as a useful flow-cytometry marker to assess TCR-αβ T-cell clonality [58,59].

In this scenario, elucidating the expression and function of CD39, CD73, and CD38 in the peripheral blood T cells of SS patients can offer new candidate markers with potential diagnostic utility and enhance our understanding of the biological significance of these molecules in SS. Among the studies included in this review, 13 studies used flow cytometry to analyze the expression of CD39, CD73, or CD38 in CTCL. These studies consistently demonstrated elevated levels of CD39 and/or CD73 on malignant T cells compared to normal T cells. The CD38 expression was variable among patients but was often associated with advanced disease stages.

#### 3.1.1. CD39 Expression

The abnormal activation of the CD39/CD73/adenosine axis in tumor tissues has been extensively documented [60,61,62]. The biochemical activity of CD39 and CD73 is closely associated with immune responses. As early as 2007, it was reported that the CD39/CD73 pathway regulates the immunosuppressive functions of Tregs. In CD39 knockout mice, CD4+ T cells exhibit a significantly reduced capacity to hydrolyze ATP, thereby failing to produce adenosine. Thus, despite the presence of other nucleotidases, CD39 is the major rate-limiting ectoenzyme of the ATP-adenosine pathway in T cells [63]. Moreover, several studies have confirmed the expression of CD39 in various tumor cells and its role in suppressing anti-tumor immune responses [64,65]. In 2019, Bensussan et al. demonstrated by CyTOF analysis that CD39 was mainly expressed by the malignant T-cell cluster in SS patients, while its expression was limited to a small number of cells in healthy donors (HD). These data were further confirmed by flow cytometry on blood samples from 20 SS patients, where the CD39 expression was significantly higher in CD4+ T cells from SS patients compared with HD. Moreover, by differentiating between malignant and non-malignant CD4+ T cells via a TCRvβ assessment, it has been shown that the majority of CD39+CD4+ cells were CD4+TCRvβ+ malignant cells, with a strong correlation with the natural killer cell receptor KIR3DL2 expression. Based on these results, CD39, in combination with KIR3DL2, was recommended as a diagnostic marker for Sézary cells [38]. Further studies by Sonigo et al., Yakymiv et al., and Battesti et al., involving 36, 11, and 37 SS patients, respectively, confirmed the high frequency of CD39+CD4+ T cells in SS [42,47,49].

The single nucleotide polymorphism (SNP) rs10748643 (A→G), in intron 1 of the human *ENTPD1*/CD39 gene, is associated with the increased frequency of CD39+ Tregs in healthy subjects. Indeed, individuals carrying the GG genotype exhibit a significantly higher number of CD39+ Tregs, and a greater capacity to suppress the production of IFN-γ and IL-17 by effector cells, compared to the AG or AA genotypes [66]. Similarly, Picozza et al. found that SS patients with the GG and AG genotype have a higher frequency of CD4+CD39+ Sézary cells compared to those with the AA genotype. Moreover, in a retrospective cohort of 47 SS patients, they found that a high CD39 expression, combined with the GG or AG genotypes, is associated with a better prognosis [40]. However, in an independent cohort of 62 SS patients from Leiden University, ENTPD1 SNP genotype failed to recognize patients at risk and to predict the disease course. SNP rs10748643 within the promoter region of ENTPD1 (the gene encoding CD39) had no prognostic value in Sézary patients [67]. The prognostic significance of CD39 as a genetic marker in SS patients deserves further investigation.

#### 3.1.2. CD73 Expression

CD73, as the second rate-limiting enzyme in purine nucleotide metabolism, plays a crucial role in the hydrolysis of AMP into adenosine, making it an indispensable component of the adenosine pathway [68]. Consequently, it is a potential target for anti-tumor therapy, with various anti-CD73 antibodies and small molecule inhibitors currently undergoing clinical trials [62]. Although there are limited data concerning the significance of CD73 in CTCL, existing studies indicate the aberrant expression of CD73 on Sézary cells. Sonigo et al. demonstrated significant heterogeneity in the expression of CD39 and CD73 on malignant CD4+ T cells in the peripheral blood of SS patients. Over half of patients’ tumor cells exhibited a CD39+CD73− phenotype, fewer than 30% were CD39−CD73+, and only a minority were double-positive (CD39+CD73+). Five out of thirty-seven tested patients displayed no overexpression of either marker on the malignant cells. Meanwhile, non-malignant CD4+ T cells and CD8+ T cells showed a trend toward a downregulated CD73 expression. The CD73 expression was upregulated in NK cells but showed no significant change in B cells [42]. Yakymiv et al. confirmed the aberrant expression of CD39 and CD73 in the CD4+ T cells from the selected SS patients. Indeed, two out of eleven SS patients analyzed exhibited a high frequency of CD73-positive CD4+CD26− T cells with an elevated MFI. The expression of both CD39 and CD73 was unaffected by the therapy administered, except in the case of the anti-CCR4 monoclonal antibody mogamulizumab treatment that drastically reduced the number of circulating Sézary cells. Furthermore, the authors demonstrated, through co-culture experiments, that both CD39 and CD73 exhibit enzymatic activity on Sézary cells, contributing to the generation of extracellular adenosine, driving the suppression of anti-tumor immune responses [47]. Overall, these preliminary findings indicate a potential involvement of CD73 in the biology of Sézary cells.

#### 3.1.3. CD38 Expression

CD38, a multifunctional ectoenzyme and signaling protein, is involved in immune regulation, metabolic control, and tumor progression. It is expressed in many immune cell types, including bone marrow progenitors, natural killer cells, monocytes, activated T and B lymphocytes, and plasma cells. CD38 is a prognostic marker in chronic myeloid leukemia, and a target in multiple myeloma immunotherapy [69,70].

The expression of CD38 in CTCL is quite heterogeneous. In 2015, a study proposed that the expression of CD38 on CD4+ T cells in a cohort of 107 SS patients was significantly lower than in HD, and CD4+CD38− cells matched the CD4+CD26− cell population [36]. Interestingly, a retrospective study in 76 SS patients found that, although most SS patient cells were CD38-negative, a subset of patients (5 out of 76, 6.6%) had more than 50% of circulating CD4+CD38+ cells, often associated with a high number of circulating Sézary cells and a high CD4/CD8 ratio [41]. Isabelle et al. analyzed 51 mature T-cell lymphomas, including 3 SS and 4 MF, by flow cytometry, and found that the CD38 expression was higher than in HD across almost all subtypes, albeit with considerable heterogeneity among patients [43]. Torrealba et al. analyzed CD8+ T cells in 17 SS patients before the initiation of treatment and found an increased expression of CD38 mainly in effector/memory cells, which correlated with the chronic activation status of CD8+ T cells in SS patients compared to the control [37]. The chronic activation of CD8+ T cells, characterized by functional exhaustion and a high expression of inhibitory receptors, leads to a significant decrease in their cytotoxic and proliferative capabilities [71]. Recent studies by Ta et al. combined flow cytometry, single-cell sequencing, and immunohistochemistry to assess the expression of CD38 on neoplastic cells in blood and skin from patients with different CTCL subtypes. In blood, multiparameter flow cytometry revealed that Sézary cells had a distinct intermediate level of CD38 expression, lower than that of benign T cells from SS patients and of CD4 T cells from HD. They also demonstrated that a higher CD38 expression on malignant T cells is associated with a poorer prognosis and shorter survival in keeping with a similar observation by Pavlova et al. [44,46]. These findings suggest the potential clinical utility of CD38 as a prognostic marker as well as a therapeutic target for advanced CTCL. However, further studies are needed in order to validate the associations between CD38 and advanced CTCL, since these results were based on a limited number of cases.

### 3.2. Expression of CD39, CD73, and CD38 in CTCL Skin Biopsies

Skin biopsy is a key step in the diagnosis of CTCL, enabling the detection of atypical T-cell infiltration in the dermis and epidermis. An immunohistochemical analysis can further identify peculiar features of the tumor cells, such as the loss of CD7, low number of CD8+ infiltrates, and increased Ki67 expression [72]. Determining the immunophenotypic profile and the distribution of CD39, CD73, and CD38 in skin can help in assessing the disease severity, providing essential information for the formulation and adjustment of therapeutic strategies.

#### 3.2.1. CD39 and CD73 Expression

The expression of CD39 and CD73 varies in the epidermis of normal skin and skin lesions. In normal skin, CD39 is expressed at low levels in the dermis and on Langerhans cells in the epidermis, whereas CD73 is expressed in both the epidermis and the dermis [15,73]. The increased expression of CD39 and CD73 is observed in various tumors, including cutaneous malignancies. Studies by Whitley et al. demonstrated a higher frequency of CD39 in the area surrounding squamous cell carcinoma lesions compared to non-lesional tissue, indicating its potential role within the TME [74,75]. In skin biopsies of patients with SS, MF, and subcutaneous panniculitis-like T-cell lymphoma (SPTL), a significant increase in CD39 expression was observed compared to patients with benign inflammatory disorders. In addition, CD39 expression was found to be correlated between the blood and skin compartments [49]. This study confirms the previous data showing that both SS patients with a high expression of CD39 or CD73 in circulating CD4+ T cells also expressed elevated levels of CD39 or CD73 in the infiltrating lymphocytes of skin biopsies, with a predominant cytoplasmic and membranous distribution of CD39, and a diffuse membranous expression with a focal dot-like distribution of CD73. Notably, this aberrant expression was already appreciable by the time of diagnosis [47]. Furthermore, Scheffschick et al. performed a flow cytometry analysis in infiltrating NK and CD8+ T cells from 24 CTCL skin biopsies and observed a similar CD73 expression on both cell populations compared to healthy skin, with no significant differences [45].

#### 3.2.2. CD38 Expression

CD38 participates in adenosine generation via the CD203a/CD73 pathway, in selected tumors such as non-small-cell lung cancer (NSCLC) and melanoma, where the increased CD38 expression contributes to increased adenosine production and immunosuppressive effects [76,77]. An immunohistochemical analysis of skin samples from 63 SS patients taken at diagnosis revealed a low or absent CD38 expression in most infiltrating lymphocytes, with only a minority of skin samples (n = 8, 10.5%) exhibiting significant CD38+ lymphocyte infiltration [41]. Subsequently, Ta et al. utilized single-cell RNA sequencing in skin biopsies from CTCL patients to demonstrate that CD38 expression levels were higher in CTCL skin lesions compared to healthy skin. Moreover, CD38 was co-expressed with multiple CTCL tumor cell markers, providing evidence for CD38 expression in CTCL tumor cells [46]. Similarly, Pavlova et al. performed a comparative analysis of skin lesions between patients with CTCL and atopic dermatitis with Nanostring technology, and found higher levels of CD38 expression in the skin lesions of CTCL patients, than in benign inflammatory skin disease [44]. Additionally, an immunohistochemical analysis in skin and lymph node samples from 52 patients with CTCL highlighted the increased CD38 expression in skin biopsies from aggressive CTCL subtypes such as SS with large-cell transformation, primary cutaneous γδT-cell lymphoma, and primary cutaneous NK/T-cell lymphoma, negatively correlating with overall survival [44,46]. Hence, CD38 holds promise as a therapeutic target for aggressive and refractory CTCL. Nevertheless, further research is required before drawing definitive conclusions on the utility of CD38 as a therapeutic target in SS.

Six studies focused on skin biopsies, employing IHC to investigate the presence and localization of CD39, CD73, and CD38 in CTCL lesions. Overall, the results indicated that these markers were predominantly expressed in the epidermal and dermal regions infiltrated by malignant T cells, providing important clues for further investigation of the role of these molecules in the TME.

### 3.3. CD39/CD73/CD38 Expression in CTCL and Therapeutic Potential

**Figure 3:**
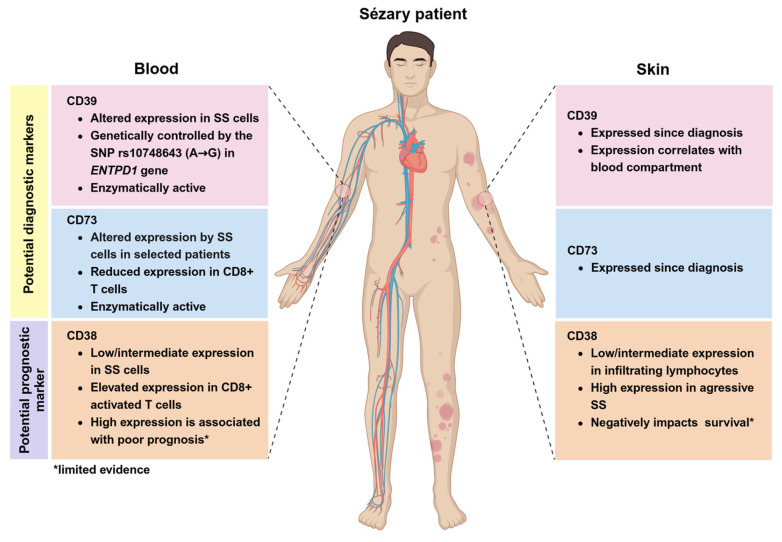
The summary of CD39, CD73, and CD38 expression in SS patient’s blood and skin.

This review comprehensively summarizes the expression patterns of CD39, CD73, and CD38 in CTCL (Figure 3), providing new insights for the diagnosis, prognosis, and treatment of aggressive CTCL including SS. Historically, ≥40% CD4+CD7− and/or ≥30% CD4+CD26− of total lymphocytes has been commonly used to identify Sézary cells. However, growing evidence suggests limitations with this approach, as it may miss certain malignant T cells, highlighting the need for more effective marker combinations to enhance the diagnostic accuracy for Sézary cells [54,78].

CD39, a key enzyme in the canonical adenosine pathway, has been shown in multiple studies to be highly expressed on malignant T cells in the majority of SS patients. A high CD39 expression is a promising marker to identify Sézary cells when used in combination with KIR3DL2 [38]. A recent study using a multi-omic systems immunology approach toward the profiling of CTCL patients from two anti-PD-1 clinical trials identified CD39, together with CD95 and KIR3DL2, as the most discriminatory markers for neoplastic T cells in leukemic CTCL patients [50]. In addition, it has been suggested that patients with a high CD39 expression apparently have better prognosis [40]. This is in line with the observation that, in triple-negative breast cancer patients, the presence of CD39+ T cells predicts an improved prognosis, and, in NSCLC, the redistribution of CD39+CD8+ T cells contributes to better clinical outcomes [79,80]. However, a high expression of CD39 helps adult T-cell leukemia/lymphoma cells escape anti-tumor immunity through the extracellular ATPDase-adenosine cascade [81]. Moreover, a correlation between CD39 expression and tumor progression was found in prostate cancer [82]. These contrasting observations underline that the relationship between CD39 expression and patient outcomes is complex and varies significantly across tumor types.

CD73 exhibits heterogeneous expression across immune cell subsets in CTCL. Preliminary evidence indicates that a high CD73 expression is often associated with a poor prognosis and lymph node metastasis in selected cancers, such as breast and ovarian cancer [83,84]. Nonetheless, the prognostic significance of CD73 in CTCL remains to be determined. Based on the data from a limited number of patients, it is tempting to predict that its variable expression might allow the identification of specific SS patient subgroups (CD73+ individuals), which has the potential to assist dermatologists in the development of personalized therapies for SS.

CD38 is a marker for multiple myeloma cells, but it is also expressed by other tumors, although at lower levels. Ibrahim et al. found CD38 expression in approximately 27–46% of chronic lymphocytic leukemia (CLL) cases, with higher expression levels associated with increased aggressiveness and a poorer prognosis [85]. Unlike CD39 and CD73, the CD38 expression in SS patients exhibits considerable heterogeneity. Most SS patients show a low/intermediate CD38 expression in CD4+ T cells, with the number of CD4+CD38− cells roughly equal to those of CD4+CD26− cells. Instead, CD38 is frequently highly expressed in CD8+ T cells of SS patients, indicating different functions across T-cell subpopulations [86]. Skin biopsies reveal that CD38 is more prevalent in aggressive and refractory CTCL patients, often correlating with a poorer prognosis. These studies indicate that the heterogeneous expression of CD38 reflects the biological characteristics of different tumor types and highlights its potential utility as a therapeutic target.

Targeted therapy has emerged as one of the leading strategies in anti-tumor treatment in recent years and targeted therapeutic agents mainly include monoclonal-antibody-based drugs and small molecule inhibitors [87]. Recently, the development of targeted therapies against key regulators of the adenosine pathway in the TME, such as CD39, CD73, and CD38, has gained increasing attention. Among monoclonal antibodies targeting CD39, TTX-030, a non-competitive CD39 inhibitor, is currently undergoing a phase I clinical trial and has shown efficacy in inhibiting CD39 in ATP-enriched TME [88]. In addition, the murine-derived monoclonal antibody B66 has shown significant anti-tumor metastatic effects in murine models by promoting the expansion and survival of NK cells [89]. The AB598 monoclonal antibody proved to be effective in suppressing tumor growth by inhibiting CD39 enzymatic activity in the MOLP8 xenograft model and it is currently undergoing Phase I/1b clinical trials, in combination with chemotherapy and anti-PD-1 therapies in solid tumors, including bladder cancer, cervical cancer, and esophageal cancer (NCT05891171) [90]. Other monoclonal antibodies targeting CD39 or CD73, such as IPH5201 and IPH5301, have been shown to effectively block the hydrolysis of ATP into adenosine. This inhibition restores T-cell activation in vitro in multiple myeloma patients and promotes anti-tumor immunity [91]. The human IgG1λ anti-CD73 monoclonal antibody oleclumab (MEDI9447) enhanced T-cell activity and demonstrated anti-tumor effects in advanced colorectal cancer, pancreatic ductal adenocarcinoma, and NSCLC in the first-in-human study [92]. In addition to monoclonal antibodies, small molecule inhibitors are promising drugs in CD39/CD73-targeted therapies. For instance, sodium polyoxotungstate (POM-1), a classical small molecule CD39 inhibitor, has been reported to exhibit anti-tumor activity in various cancers, including multiple myeloma, acute myeloid leukemia, and bladder cancer [21,93,94]. The combination of POM-1 with CD73 small molecule inhibitors and the A2A receptor antagonist AZD4635 blocks the adenosine pathway, promoting IFN-γ production, activating immune cells, and reducing Tregs [21]. Furthermore, the protein kinase inhibitor ceritinib (Zykadia), an approved treatment for ALK-positive NSCLC, is a non-competitive inhibitor of CD39. It blocks the conversion of ATP to adenosine and has shown efficacy in triple-negative breast cancer and melanoma [95]. AB680 is the first CD73 small molecule inhibitor to enter clinical development. Preclinical studies have shown that it can effectively restore T-cell proliferation and demonstrate good safety and efficacy in PDAC, colorectal cancer, and melanoma [96,97,98]. These findings suggest that therapies targeting CD39 and CD73 contribute to the restoration of anti-tumor immunity and show preliminary effectiveness.

Simultaneously, research on combination therapies targeting CD39 and CD73 with immune checkpoint inhibitors is thriving. A study suggests that the enrichment of CLA+CD39+ Tregs in anti-PD-1-resistant CTCL patients may contribute to treatment resistance [50]. Another preclinical study has shown that SRF617, a fully human anti-CD39 antibody, synergizes with PD-1 checkpoint inhibitors to enhance anti-tumor efficacy and improve overall survival [99]. Currently, a phase II trial (NCT06119217) is ongoing to assess the efficacy of TTX-030 combined with chemotherapy and budigalimab (an investigational anti-PD-1 antibody) in the treatment of metastatic PDAC, while the combination of oleclumab (anti-CD73 mAb) and durvalumab (anti-PD-1 mAb) has shown improved clinical responses in PDAC and advanced colorectal cancer [92]. PT199 is a CD73 antibody with a unique mechanism of action. Unlike other antibodies, PT199 is capable of fully inhibiting both soluble and membrane-bound CD73. Currently, clinical trials are underway to evaluate PT199 in combination with PD-1 inhibitors for the treatment of locally advanced or metastatic solid tumors (NCT05431270) [100]. These findings prompt speculation that CD39 and CD73 may represent potential therapeutic targets in CTCL, and suggest that combining the targeting of the adenosine pathway, along with immune checkpoint inhibitors, may have synergistic anti-tumor effects and improve patient survival.

CD38 is increasingly emerging as a potential target for anti-tumor therapies. Daratumumab, the first monoclonal antibody targeting CD38, has been approved for use with standard myeloma regimens. It stimulates effector CD8+ T-cell proliferation and enhances anti-tumor immune responses [101]. Alongside daratumumab, isatuximab is also used in the treatment of multiple myeloma. Both antibodies exploit Fc-dependent immune effector mechanisms, such as antibody-dependent cellular cytotoxicity, complement-dependent cytotoxicity, and antibody-dependent cellular phagocytosis, to directly kill tumor cells and exert anti-tumor effects [34,102]. Moreover, bispecific antibodies VP301 and small molecule inhibitors N1-inosine 5′-monophosphate (N1-IMP) have shown potential in enhancing therapeutic efficacy and overcoming resistance. For instance, VP301 targets both CD38 and ICAM-1, ensuring effectiveness even in tumors with a low CD38 expression [103]. While N1-IMP significantly suppresses CD38 enzymatic activity and boosts immune responses [104]. As CD38-targeted therapies continue to evolve, various other CD38-targeted therapies are in the preclinical or clinical development stages [105,106]. Consequently, the heterogeneous expression of CD38 presents both a challenge and an opportunity for the development of personalized therapies in CTCL.

Nonetheless, when conventional markers fail to accurately identify all Sézary cells, the high expression of CD39 and/or CD73 in SS patients offers new potential diagnostic markers, assisting in the identification of Sézary cells. The inclusion of CD38 can further aid in recognizing disease progression stages, assessing patient prognosis, and helping clinicians better evaluate the tumor burden. The use of monoclonal antibodies and small molecule inhibitors targeting CD39, CD73, and CD38, or with immune checkpoint inhibitors, holds promise as a therapeutic option for SS, providing new directions and guidance for the treatment of patients.

## 4. Conclusions

In summary, this study systematically reviews the existing literature to reveal the expression patterns and potential roles of CD39, CD73, and CD38 in CTCL and SS, in particular. These ectoenzymes are not only good candidates as diagnostic markers but also as potential therapeutic targets [107]. Future research should further investigate the specific mechanisms and clinical applications of these molecules, aiming to provide more precise diagnostic tools and effective treatment options for patients with CTCL and SS.

## Figures and Tables

**Table 1:** Studies included in the review and related findings. (FC, flow cytometry; IHC, immunohistochemistry; * review; ** commentary).

No.	Author/Year/DOI	CD39/CD73/CD38	FC/IHC	Main Finding
1	Novelli, M., et al. (2015) https://doi.org/10.1309/AJCP1NA3YCHCDEIG [36]	CD38	FC	In SS, CD38 expression was lower than HD, and CD4+CD38− cells matched with the CD4+CD26− cells population.
2	Torrealba, M. P., et al. (2018) https://doi.org/10.18632/oncotarget.23334 [37]	CD38	FC	In SS, CD38 expression increased in effector/memory CD8+ cells and high serum levels of sCD38 were detected.
3	Bensussan, A., et al. (2019) https://doi.org/10.1016/j.jid.2018.09.026 [38]	CD39	FC	In SS, CD39 expression was higher than HD and CD39 with KIR3DL2 was recommended as a diagnostic marker for Sézary cells.
4	Cristofoletti, C., et al. (2022) https://doi.org/10.3390/cancers14122847 [39]	CD39	*	Highlighting CD39 and PD-1 as new genetic markers for SS.
5	Picozza, M., et al. (2022) https://doi.org/10.1016/j.jid.2022.04.017 [40]	CD39	FC	In SS, individuals carrying the A/G–G/G genotype showed a higher frequency of clonal CD4+CD39+ Sézary cells and high CD39 expression correlated with a better prognosis.
6	Quaglino, P., et al. (2022) https://doi.org/10.1155/2022/3424413 [41]	CD38	FC/IHC	In SS, CD38 expression was predominantly negative on both blood and skin, but varied across patients.
7	Sonigo, G., et al. (2022) https://doi.org/10.1182/blood.2021014782 [42]	CD39/CD73	FC	In SS, significant heterogeneity in the expression of CD39 and CD73 on malignant CD4+ T cells.
8	Isabelle, C., et al. (2023) https://doi.org/10.1182/bloodadvances.2023009807 [43]	CD38	FC	In mature T-cell lymphomas including SS (3/51), CD38 expression on the malignant T cells was higher than CD3+ T cells from HD.
9	Pavlova, O., et al. (2023) https://doi.org/10.1016/j.jid.2023.02.011 [44]	CD38	FC/IHC	In SS blood, higher CD38 expression on malignant T cells was associated with poorer prognosis. In CTCL skin, CD38 expression was higher than atopic dermatitis.
10	Scheffschick, A., et al. (2023) https://doi.org/10.3389/fimmu.2023.1168684 [45]	CD73	FC/IHC	In CTCL skin biopsies, CD73 expression on NK and CD8+ T cells was comparable to healthy skin, with no significant differences.
11	Ta, V. A., et al. (2023) https://doi.org/10.1016/j.jid.2023.01.009 [46]	CD38	FC/IHC	In SS blood, Sézary cells showed intermediate CD38 expression. In CTCL skin including SS (6/14), CD38 expression was higher than in healthy skin.
12	Yakymiv, Y., et al. (2023) https://doi.org/10.1182/blood.2022017259 [47]	CD39/CD73	FC/IHC	In SS blood, CD39 and CD73 exhibited aberrant expression on CD4+ T cells and enzymatic activity on Sézary cells. In the SS skin infiltrating lymphocytes, CD39 and CD73 expression was elevated, and corresponded to increased levels on blood CD4+ T cells.
13	Whittaker, S., et al. (2023) https://doi.org/10.1182/blood.2022018185 [48]	CD39/CD73	**	The findings of Yakymiv et al., highlighted that the CD39/CD73/adenosine pathway contributes to immuno-suppression in SS.
14	Battesti, G., et al. (2024) https://doi.org/10.1002/ski2.334 [49]	CD39	FC/IHC	In SS, CD39 overexpression by malignant and non-malignant T cells and CD39 expression was correlated between the blood and skin compartments.
15	Glass, D. R., et al. (2024) https://doi.org/10.1016/j.xcrm.2024.101527 [50]	CD39	FC	In CTCL, CD39, CD95, and KIR3DL2 were recommended as the most discriminatory markers for neoplastic T cells.

## Data Availability

No new data were created or analyzed in this study. Data sharing is not applicable to this article.
